# Light-induced translocation of cyclic-GMP phosphodiesterase on rod disc membranes in rat retina

**Published:** 2008-12-29

**Authors:** Jing Chen, Tatsuro Yoshida, Mark W. Bitensky

**Affiliations:** Department of Biomedical Engineering, College of Engineering, Boston University, Boston, MA

## Abstract

**Purpose:**

Cyclic GMP phosphodiesterase (PDE) is the light-regulated effector enzyme of vertebrate rods. Upon photo-activation of rhodopsin followed by activation of transducin/GTP, PDE rapidly hydrolyzes 3′, 5′-cyclic GMP (cGMP) to 5′-GMP, which results in closure of cGMP-dependent ion channels and generation of a nerve signal. In the rod photoreceptors, PDE is entirely localized within the rod outer segment (ROS), a specialized compartment consisting of thousands of disc stacks. This study investigated the effects of light on the subcellular localization of PDE in ROS.

**Methods:**

Adult rats were either dark- or light-adapted for various durations before eyes were isolated and processed for transmission electron microscopy. Immunogold electron microscopy was performed with antibodies against PDE. Lateral displacement of PDE on ROS disc membrane was analyzed from electron micrographs. PDE enzymatic activities were measured with thin layer chromatography.

**Results:**

Light exposure induced translocation of PDE away from the edges of the dark-adapted disc membranes adjacent to the ROS plasma membrane. In dark-adapted ROS, a substantial portion (19±2%) of total PDE was localized near the edges of the disc membranes. Within 1 min of light exposure in the presence of GTP, over half of such PDE molecules (10±1% of total PDE) had moved away from the edges of the discs toward disc center. This light induced translocation of PDE was GTP dependent, as the effect was abolished when hydrolysis-resistant GTPγS was used in place of GTP. The percentage of PDE found near the disc edge corresponds to the fraction of PDE activity relative to maximal PDE activity revealed by limited trypsin proteolysis.

**Conclusions:**

These results suggest that light and GTP modulates lateral displacement of PDE, which might contribute to light-induced reduction of rod photoreceptor sensitivity.

## Introduction

The vertebrate visual system can adjust its sensitivity over a wide range of light intensities, a capacity of fundamental importance for vision and species survival. Light encountered in vertebrate habitats on the surface of this planet can vary by many orders of magnitude during a normal day and night cycle. While a large fraction of visual adaptation is accomplished by simply switching between cone (bright light) and rod (dim light) vision, several additional molecular and cellular processes contribute to light-adaptation pathways in both rods and cones [1-4]. Among these mechanisms, the translocation of molecules involved in visual signaling between rod outer and inner segments has been proposed recently as a primary mechanism of light adaptation in rod photoreceptors [5-9].

The rod outer segment (ROS) is a cylindrical structure containing a stack of disc membranes. The visual signal transduction proteins, including rhodopsin, transducin (G_t_), and cyclic guanosine monophosphate (cGMP) phosphodiesterase (PDE), are localized on these discs. The visual signaling cascade begins with photon absorption by rhodopsin. Photo-activated rhodospin in turn activates G_t_, the heterotrimeric guanosine triphosphates (GTP) binding protein of the rod photoreceptor cell [10,11], and facilitates the exchange of GTP for GDP on the G_tα_ subunit. G_tα_-GTP then interacts with and activates PDE. Rod PDE is composed of two catalytic subunits, α (98 kDa) and β (97 kDa), and a pair of identical inhibitory γ subunits (10 kDa) [12,13]. Interaction with G_tα_-GTP relieves the inhibitory PDE γ subunits, thereby activating PDE [14,15]. Activated PDE rapidly hydrolyzes cGMP to 5′ GMP, resulting in closure of the cGMP-gated cation channels in the ROS plasma membrane and generation of membrane potential [16-18].

PDE has generally been assumed to be randomly distributed on ROS disc membranes ever since it was first described [19-23]. However, if activated PDE is localized in areas of the disc membrane in proximity to the plasma membrane where ion channels are located, it may be in an optimal position to effect rapid decrease of the cGMP concentration and ensure fast closure of the ion channels. Direct interaction of PDE with cGMP dependent channels was suggested previously [24-26], although earlier studies using immunohistochemistry and light microscopy did not provide enough resolution to explore the precise localization of PDE on ROS disc membrane surface [22,23,27].

The present study was undertaken to characterize the effects of light on the subcellular distribution of rod PDE using immunoelectron microscopy. Here we describe light- and GTP-dependent centripetal migration of PDE on ROS disc. We found that in dark-adapted rods PDE was more concentrated near the rim region of the ROS disc membranes adjacent to the ROS plasma membrane. Light exposure, in the presence of GTP, induced translocation of PDE away from the disc rim region (as well as the plasma membrane) toward the center of the discs. This light-induced PDE translocation was abolished when GTPγS replaced GTP. Postulated functional consequences of PDE translocation in the regulation of rod photoreceptor sensitivity are discussed.

## Methods

### Animals

Long-Evans rats were purchased from the Charles River Laboratory (Wilmington, MA). All procedures described in this study were approved by Boston University IACUC and conform to the recommendations of the Association for Research in Vision and Ophthalmology policy statement, regarding the care and use of animals in vision research.

### Materials

PDEα and PDEγ antibodies (rabbit) were purchased from Affinity Bioreagents, Inc. (Golden, CO). Donkey anti-rabbit secondary antibody (conjugated with 12 nm gold particles) was purchased from the Jackson Immunoresearch Laboratories Inc. (West Grove, PA). Materials for electron microscopy (EM), including glutaraldehyde, formaldehyde, uranyl acetate, LR White embedding medium, and copper grids, were purchased from Ted Pella Inc. (Redding, CA). GTP and GTPγS were purchased from Roche Applied Science (Indianapolis, IN). [8-^3^H] cGMP was purchased from PerkinElmer Inc. (Wellesley, MA). Polyethylenimine cellulose thin-layer chromatography (TLC) plates were purchased from VWR (West Chester, PA). All other chemicals were from Sigma-Aldrich (St. Louis, MO).

### Animal tissue preparation

Two-month-old female Long-Evans rats, weighing about 200 g, were maintained on a 12 h:12 h light:dark cycle. Prior to each experiment, rats were either dark-adapted overnight or light-adapted under room light (approximately 500 Lux) for at least 2 h. All subsequent procedures were performed in room light for light-adapted samples or under infrared light for dark-adapted samples using infrared image converters, until after fixation was complete. Retinal tissue was processed for EM as described in the following section. Alternatively, retinas were dissected and ROS isolated for enzymatic assays.

### Isolation of ROS

Crude ROS were isolated from dark-adapted rats as described previously [28]. Briefly, retinas were dissected and collected in ice-cold Ringer’s buffer (120 mM NaCl, 3.5 mM KCl, 10 mM HEPES, 0.2 mM CaCl_2_, 0.2 mM MgCl_2_, 10 mM glucose, 1 mM DTT, and 0.2 mM PMSF, pH 7.4). Samples were vortexed using a series of 15 bursts, each lasting 3 s. Retinal fragments were allowed to settle for 3 min and supernatant removed as crude ROS suspension. Fresh buffer was added to resuspend the settled retinal fragments, and the entire procedure was repeated 3 times. The resulting supernatants containing ROS were pooled together for experiment. To permeabilize ROS plasma membranes, samples were passed 20 times through a 27 gauge needle. The effectiveness of permeabilization was verified with addition of exogenous trypsin, which could activate PDE in permeabilized but not in intact ROS. ROS preparations were either used to measure PDE enzymatic activity or they were processed for electron microscopy. For EM analysis, dark-adapted ROS samples were incubated with either 0.5 mM GTP, 0.5 mM GTPγS, or with no nucleotides for 5 min at room temperature. Samples were then kept in the dark or exposed to room light (approximately 500 Lux) for different time duration, followed by fixation.

### Immunoelectron microscopy

Immediately after light or dark adaptation, 2% paraformaldehyde and 2% glutaraldehyde in 0.1 M sodium phosphate buffer (PB), pH 7.4, was injected between the lens and retina to enable fixation of the whole eyecup. Rats were sacrificed using CO_2_ narcosis followed by cervical dislocation. Eyes were rapidly enucleated, the cornea and lens removed, and eyecups incubated in the above fixative for an additional 1 h at room temperature. After washing in PB, fixed eyecups were cut into small fragments, dehydrated in 50%, 70%, and 90% ethanol, embedded in LR White medium (Ted Pella Inc.; Redding, CA), and polymerized at 65 °C for 48 h. Samples from isolated ROS were processed similarly with fixation steps, embedded in agar, cut into small pieces, dehydrated and embedded in LR White resin. Ultrathin sections (50–100 nm) were cut on a Reichert Ultracut microtome using a diamond knife and collected on copper grids.

### Post-embedding immunogold labeling

Ultrathin sections mounted on grids were incubated on drops of PB with 10% donkey serum (Sigma) for 10 min at room temperature, followed by 3 min of incubation in PB with 20 mM glycine to react with free aldehyde groups. Grids were washed in PB, then incubated overnight at 4 °C with primary antibody (PDEα or PDEγ antibodies) diluted in PB with 0.1% BSA (PB/BSA) followed by secondary donkey anti-rabbit IgG conjugated with 12 nm gold particles for 1 h at room temperature. After extensive washing with deionized water, grids were lightly stained with 1% uranyl acetate (5 min) and viewed under a Joel JEM-2010 electron microscope (Joel Ltd., Tokyo, Japan). Electron micrographs were taken using Kodak 4489 EM (Kodak, Rochester, NY) film. In the control sections, grids were incubated with pre-immune serum without primary antibody.

### Analysis of PDE distribution

**Figure 1:**
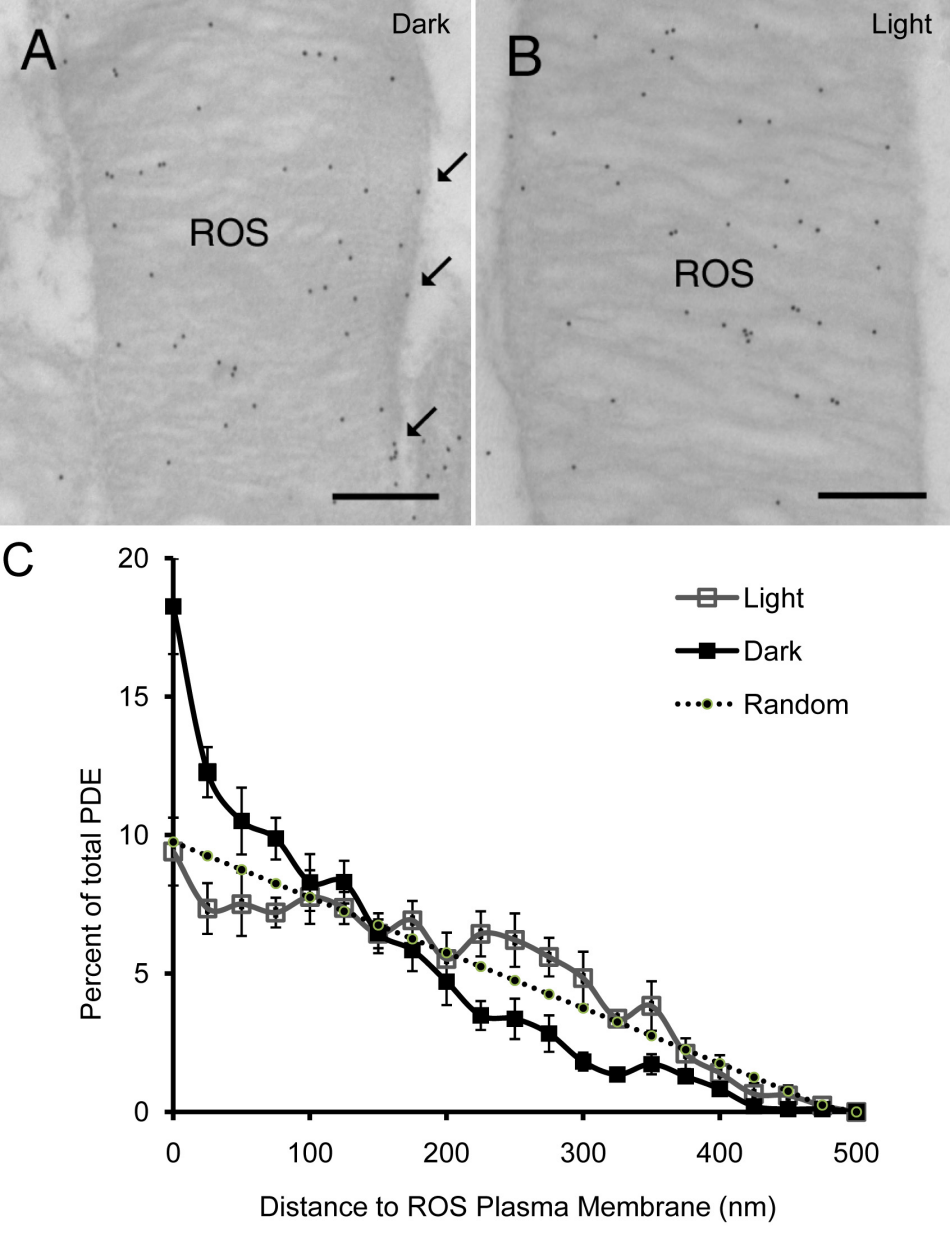
Immunogold labeling of PDE in ROS of rat retina. Light- or dark-adapted retinas were fixed and embedded in LR White resin. Ultrathin sections of the retina were incubated with PDEα antibody followed by donkey anti-rabbit secondary antibody conjugated with 12 nm gold particles. Electron micrographs were captured from dark-adapted (**A**) or light-adapted (**B**) retina. The scale bar represents 300 nm. The arrow indicates rim positioned phosphodiesterase (PDE). **C:** PDE distribution in light- or dark-adapted rod outer segment (ROS) disc membranes were quantified. Dark-adapted and light-adapted retinas were fixed and processed for immunogold labeling with PDEα antibody. The distances of each gold particle to the nearest ROS plasma membrane were measured, and the cumulative distribution profiles were plotted. Open squares represent the average distribution of PDE in light-adapted ROS, and filled squares represent PDE distribution in dark-adapted ROS. Error bars represented the standard error of the mean from four different experiments. Filled small circles with a dotted line represent a simulated random distribution of PDE. The observed distribution of PDE near ROS center in both light and dark-adapted conditions is lower than the given random distribution because the probability of obtaining sections that intersects the longitudinal axis of ROS is low.

For each experimental group, at least four animals were used. A minimum of 10 electron micrographs were taken per eye. For whole retina, sections were made in the longitudinal direction aligned to the long axis of ROS. For isolated ROS, cells with longitudinal sections were selected for quantification. Considering that the average diameter of rat ROS is roughly 1 μm, only the most central longitudinal sections of ROS with diameters greater than 600 nm were used for analysis. For each eye, at least 20 such rod cells were randomly selected for quantification of PDE localization. Distance from each gold particle to the nearest ROS plasma membrane was measured. Data were compiled in a histogram with a bin size of 25 nm. The fraction of total PDE in each bin and the standard error of the mean (SEM) were calculated. PDE molecules were scored as “near the disc edge” if the distance from the gold particle to plasma membrane was equal to or less than 25 nm. For comparison with the observed PDE distribution, a theoretical random distribution of PDE was simulated. In this simulation, a cross section of ROS is modeled as a round disk with radius *R*, and divided into *n* rings with the equal width *w*=*R/n*. Assuming a uniform distribution of PDE molecules on the ROS disc, the probability of PDE in the *i^th^* ring is the ratio of the ring’s area divided by the total area of the disk:

$$P(i)=\int_{(i-1)w}^{iw}\frac{2(R-l)}{R^{2}}dl=\frac{\left(2Rw+w^{2}\right)}{R^{2}}-\frac{2w^{2}i}{R^{2}}$$,

where *i*=1,2,…,*n*; with the first ring being closest to the disc edge and *n*th ring in the center of disc, *l* representing the distance to ROS plasma membrane. Such a simulated random PDE distribution is inversely proportional to the distance of the PDE molecules to ROS plasma membrane. The simulation in Figure 1 was generated with n=20, and *R*=500 nm, the average ROS radius measured from EM micrographs.

### PDE activity assay

PDE activity was measured with TLC using [8-^3^H] cGMP as a substrate [29]. Briefly, dark-adapted ROS were suspended in PDE activity buffer, which contained 50 mM Tris, pH 7.4, and 5 mM MgCl_2,_ at approximately 100 μg/ml (total ROS protein). ROS plasma membranes were permeabilized by passing them 20 times through a 27-gauge needle. This procedure sufficiently permeabilized ROS plasma membrane to allow PDE activation by trypsin, since the extent of trypsin activation of PDE after this procedure was similar to the maximal level of trypsin activation obtained by mild sonication of ROS for up to 30 s. The permeabilized ROS suspension was divided into four experimental groups: a) no added nucleotides; b) 0.5 mM GTP; c) 0.5 mM GTPγS; and d) 2 μg/ml trypsin (approximately 9,000 U/mg protein). For the trypsin group, PDE was pre-activated by trypsin for 5 min at room temperature followed by fivefold molar excess of soybean trypsin inhibitor to stop the trypsin activation. For light-exposed ROS, PDE activity was initiated by exposure to room light with 5 mM cGMP mixed with [8-^3^H] cGMP (final activity of 0.4 Curie/mol cGMP). All samples were rapidly mixed by vigorous agitation on a vortex mixer. At 0, 1, 2, 3, and 4 min, aliquots of reaction mixture were removed and PDE activity quenched by heating in a 100 °C paraffin bath for 1 min. For dark-adapted ROS, identical procedures were performed under infrared light. Quenched samples were centrifuged at 10,000x g for 5 min, and supernatants were analyzed by polyethylenimine cellulose thin-layer chromatography. After developing the TLC plate in 0.5 M LiCl, cyclic GMP and 5′GMP spots were identified under ultraviolet (UV) light, excised, and nucleotides were eluted in 25 mM MgCl_2_ for 15 min. Radioactivity was quantified in a Beckman LS 5000TA (Beckman Coulter, Inc., Fullerton, CA) scintillation counter.

### Statistical analysis

Results are presented as mean±SEM for animal studies and mean±SD for the non-animal studies. ANOVA with α=0.05 was used for processing the data. Two-sample *t*-tests and χ^2^-square tests were used as post tests unless otherwise indicated.

## Results

### Light-dependent PDE translocation in intact retina

Tissue sections from dark- (Figure 1A) or light-adapted (Figure 1B) intact rat retinas were immunolabeled with antibody against the α-subunit of PDE. Low magnification EM confirmed that the PDE labeling was located exclusively in the ROS (data not shown). PDE labeling was totally abolished when pre-immunization serum replaced primary antibody (data not shown). Average labeling density of PDE antibody was similar for both light- and dark-adapted samples (48.1±3.4 and 44.1±3.9 gold particles per square μm for light- and dark-adapted samples, respectively, p>0.05), indicating that the antibody recognized PDE equally well under both conditions. In the dark-adapted retina, more PDE was located near disc edges than in the light-adapted ROS (arrows in Figure 1).

**Figure 2:**
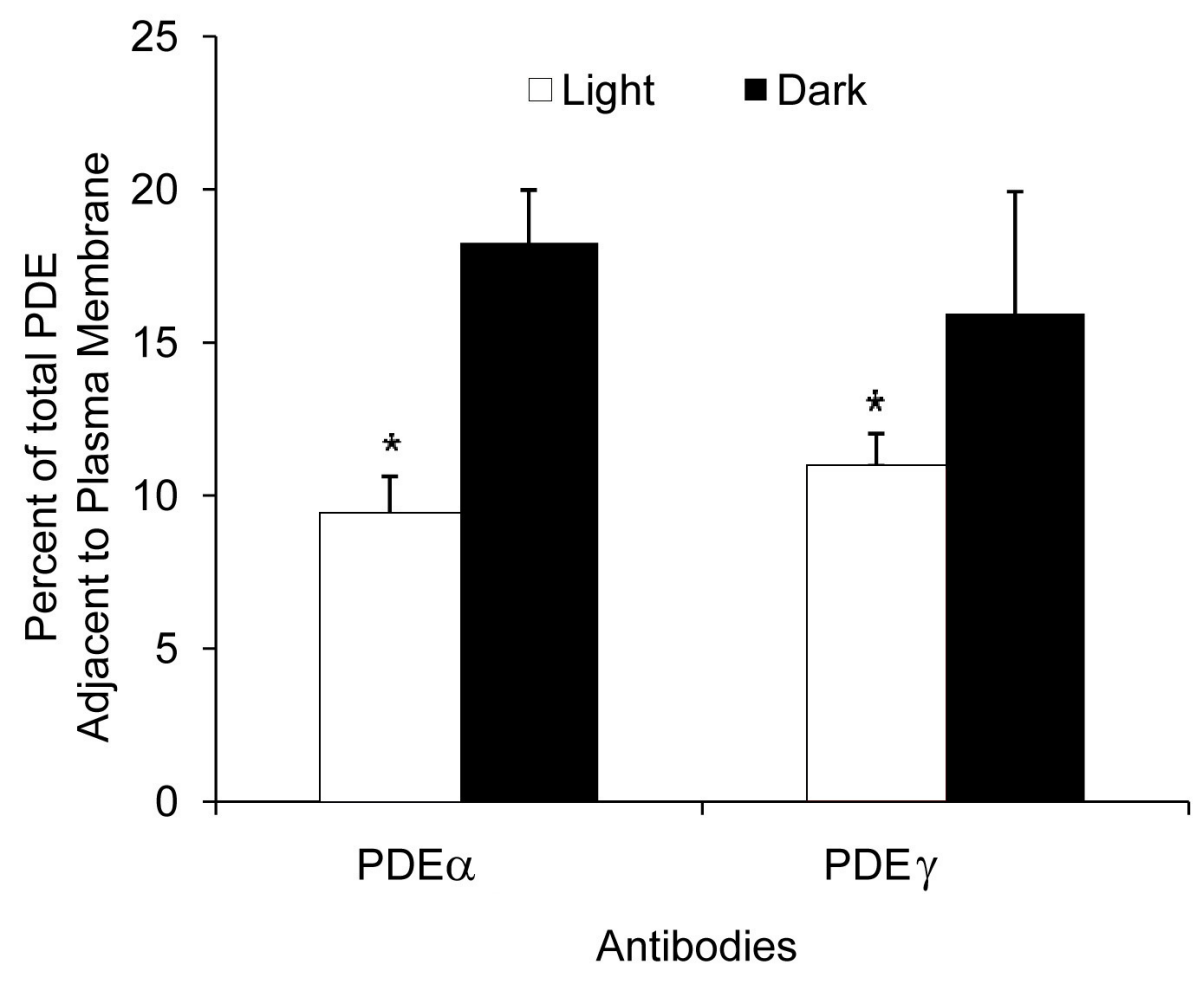
Percentage of phosphodiesterase adjacent to the rod outer segment plasma membrane measured with two different phosphodiesterase (PDE) antibodies. Retinal sections were processed for immunogold labeling using PDEα and PDEγ antibodies, respectively. Distribution profiles of PDE in light- and dark-adapted retinas were analyzed with each antibody. The percentage of total PDE immediately adjacent to the plasma membrane (within 25 nm) was plotted. The asterisk indicates a p≤0.002.

To analyze the radial distribution of PDE on rod disc membrane, we measured distances from the center of each gold particle to the nearest ROS plasma membrane. In dark-adapted rat retinas, PDE was more concentrated in the region adjacent to the disc edge near the ROS plasma membrane. For comparison, a random distribution of PDE was numerically simulated (Figure 1C, dotted line), predicting a linear relationship between the percentage of total PDE and the distance from the plasma membrane. The observed distribution of PDE in the dark-adapted samples clearly deviates from the predicted random distribution, suggesting a non-random, skewed localization of PDE toward the rim on dark-adapted disc membrane. Illumination caused movement of PDE away from the disc rim (i.e., away from plasma membrane) toward the center of the discs, resulting in a distribution of PDE closely resembling the simulated random distribution (p>0.9; not statistically significant). In the dark, an estimated 19±2% of the total PDE was located within 25 nm of the ROS plasma membrane. Illumination significantly reduced this number by more than 50% to 9±1% (Figure 2; n=4 per group; p≤0.002). These results were confirmed by experiments repeated four times using eight different animals in all. As a control, we examined transducin using G_tα_ antibody in a similar experiment, and did not observe light-induced lateral movement of the transducin (data not shown).

### Verification of PDE translocation with PDEγ antibody

In addition to PDEα antibody, we also examined the retinal sections with a second antibody directed against the γ subunit of PDE. Figure 2 illustrates the effects of light on PDE localization in retina exposed to PDEα and PDEγ antibodies. Gold particles that were within 25 nm of the ROS plasma membrane were considered to be in the rim region of the disc. Both PDE antibodies showed similar light-mediated distribution patterns. That is, in unilluminated retina, labeling of PDE was concentrated near the edge or rim of the disc membrane. With the PDEγ antibody, illumination reduced the numbers of PDE molecules in the rim region from 16±4% to 11±1%, a reduction of more than 30%, confirming the result observed using PDEα antibody (Figure 2). The results with the PDEγ antibody were slightly less marked than the results obtained with PDEα antibody. This difference may be attributed to the transducin/PDEγ interaction during light activation partially interfering with PDEγ antibody labeling. For all remaining experiments, PDEα antibody was used unless otherwise specified.

### Time course of PDE translocation in isolated ROS

**Figure 3:**
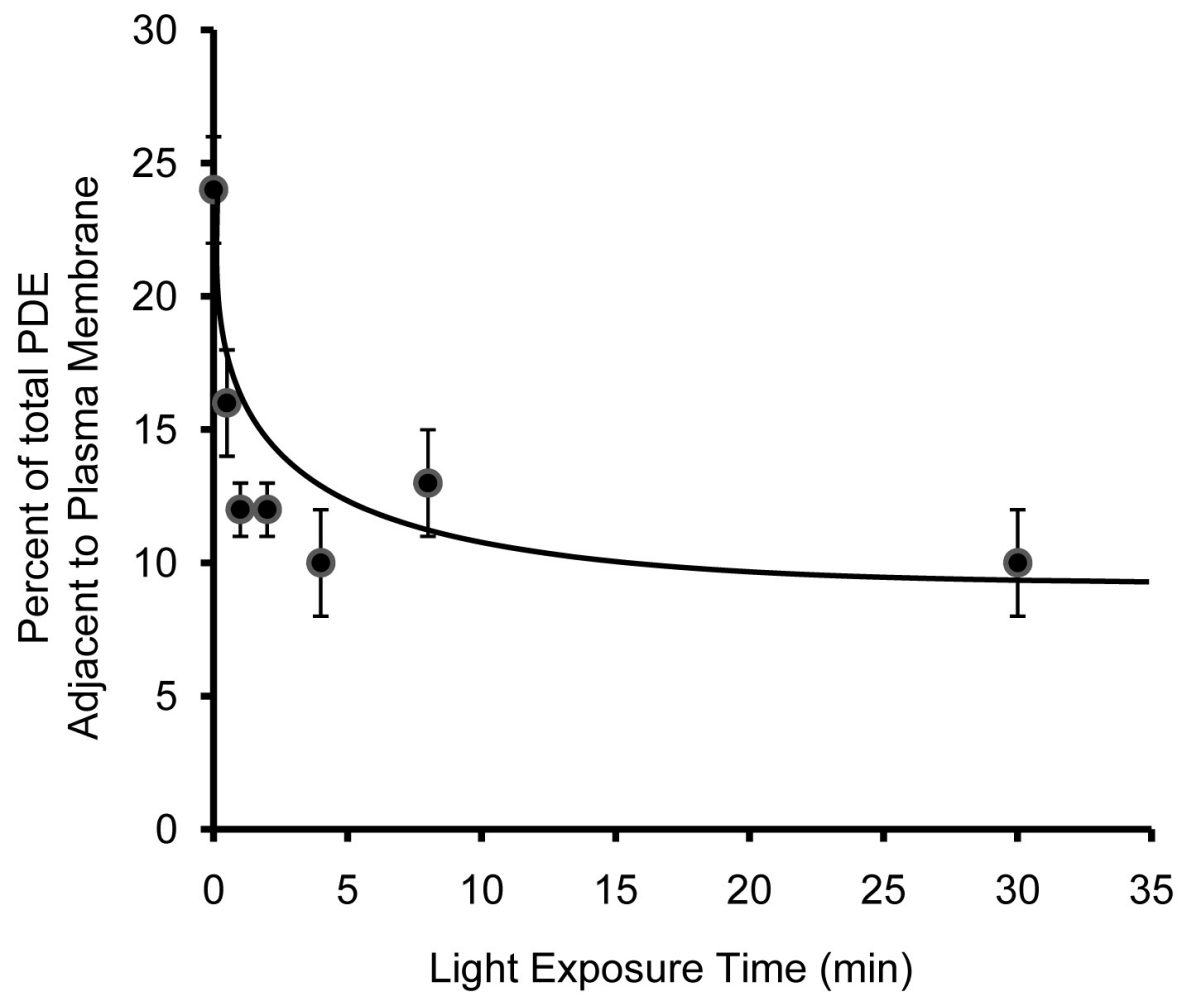
Time course of phosphodiesterase translocation. Rod outer segment (ROS) isolated from dark-adapted rats were exposed to room light (approximately 500 Lux) for various lengths of time, followed by fixation and preparation for immunogold electron microscopy. Percentage of total phosphodiesterase (PDE) adjacent to the plasma membrane (within 25 nm) was measured and plotted (p≤0.002 for 0 versus 1 min).

To investigate the kinetics of PDE movement, we explored the time course of light-induced PDE translocation. ROS were isolated from dark-adapted animals and kept either in the dark or exposed to steady room light (about 500 Lux) for 30 s, 1, 2, 4, 8, or 30 min. In the dark, 24±2% of the PDE molecules were located in the rim region (within 25 nm of the ROS plasma membrane). After 30 s of illumination, the percentage of total PDE in the rim region decreased to 16±1%. With 1 min of illumination, the number dropped to 12±1%, statistically indistinguishable from the value observed after 30 min illumination (10±2%, Figure 3). This number is also similar to the value in simulated random distribution (Figure 1C). These data indicated that light-induced translocation of PDE was a relatively rapid process that was well underway within 30 s of illumination, and was near or at completion after as little as 4 min of illumination.

### Effect of GTP and GTPγS on PDE translocation in permeabilized ROS

**Figure 4:**
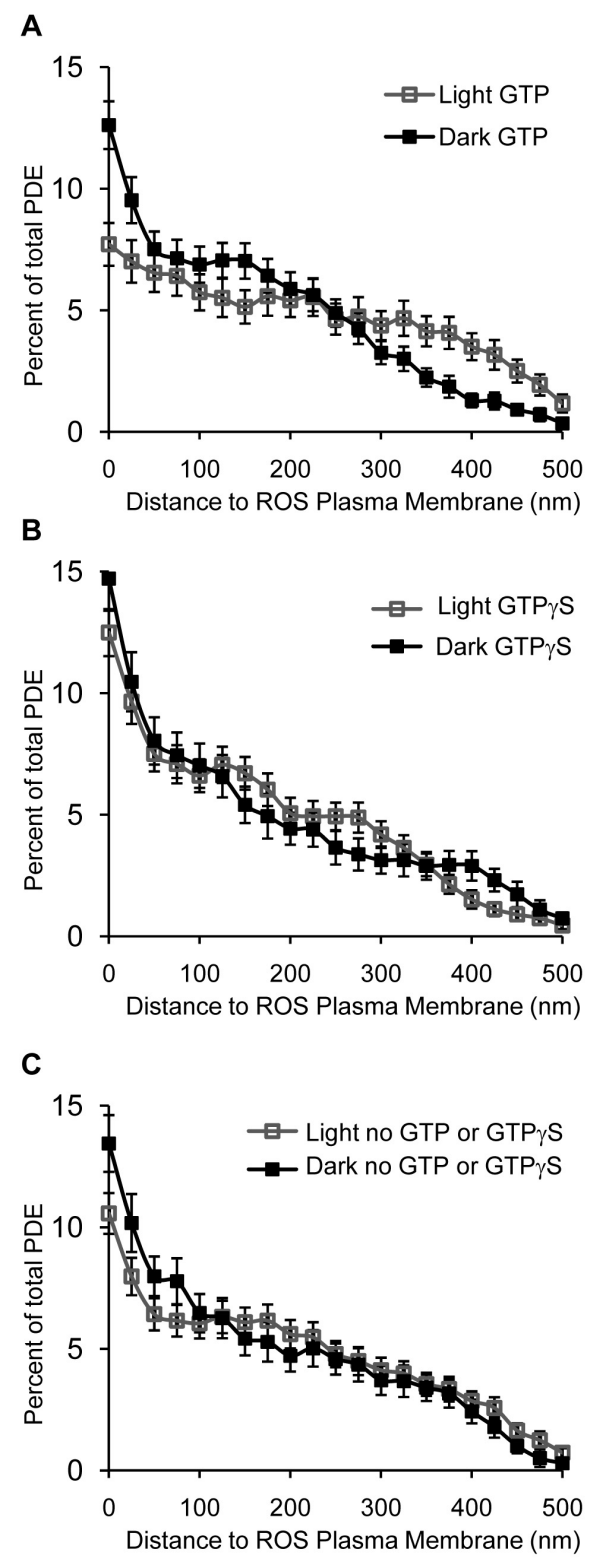
Effects of guanine nucleotides on PDE localization. Rod outer segment (ROS) from dark-adapted rats were isolated and permeabilized as described in Methods and subsequently mixed with either 0.5 mM GTP, 0.5 mM GTPγS, or no nucleotide. ROS suspensions were kept either in the dark or were exposed to room light for 30 min, followed by fixation and preparation for immunogold electron microscopy with PDEα antibody. The distances of each gold particle from the nearest ROS plasma membrane were measured, and the distribution profile of phosphodiesterase (PDE) was plotted. Panels **A** and **B** represent PDE distribution in both light- and dark-adapted conditions with guanosine triphosphates (GTP) and GTPγS, respectively. Panel **C** represents the distribution of PDE without additional nucleotides.

To investigate whether the observed PDE translocation is depended on the activation status of PDE, we compared the effects of GTP and GTPγS (a hydrolysis resistant analog of GTP) on the light-induced PDE translocation. ROS were isolated from dark-adapted animals and, after membrane permeabilization, were either kept in the dark or exposed to ambient light (about 500 Lux) for 30 min at room temperature with 0.5 mM GTP, GTPγS, or with no additional guanine nucleotides. PDE labeling densities were comparable among all groups, indicating that the antibody recognized PDE equally well under different light and guanine nucleotide status (data not shown). ROS disc membrane suspensions with added GTP showed the largest light-dependent movement of PDE away from the disc edge. In the dark with GTP, a significant population of PDE molecules was located within 25 nm of the plasma membranes. Illumination of such disc membrane suspensions caused migration of PDE molecules away from plasma membrane and toward the center of the disc (Figure 4A), similar to the centripetal translocation observed with intact retina (Figure 2). The addition of GTPγS in the presence of light prevented PDE translocation and exhibited a similar distribution as that in the dark-adapted retina (Figure 4B). Without added guanine nucleotides (in both dark and light), PDE distributions in permeabilized ROS were virtually indistinguishable from those of dark intact retina (Figure 4C), indicating that the light-induced centripetal movement of PDE requires the presence of hydrolysable GTP.

### PDE activity measurements in permeabilized ROS

**Figure 5:**
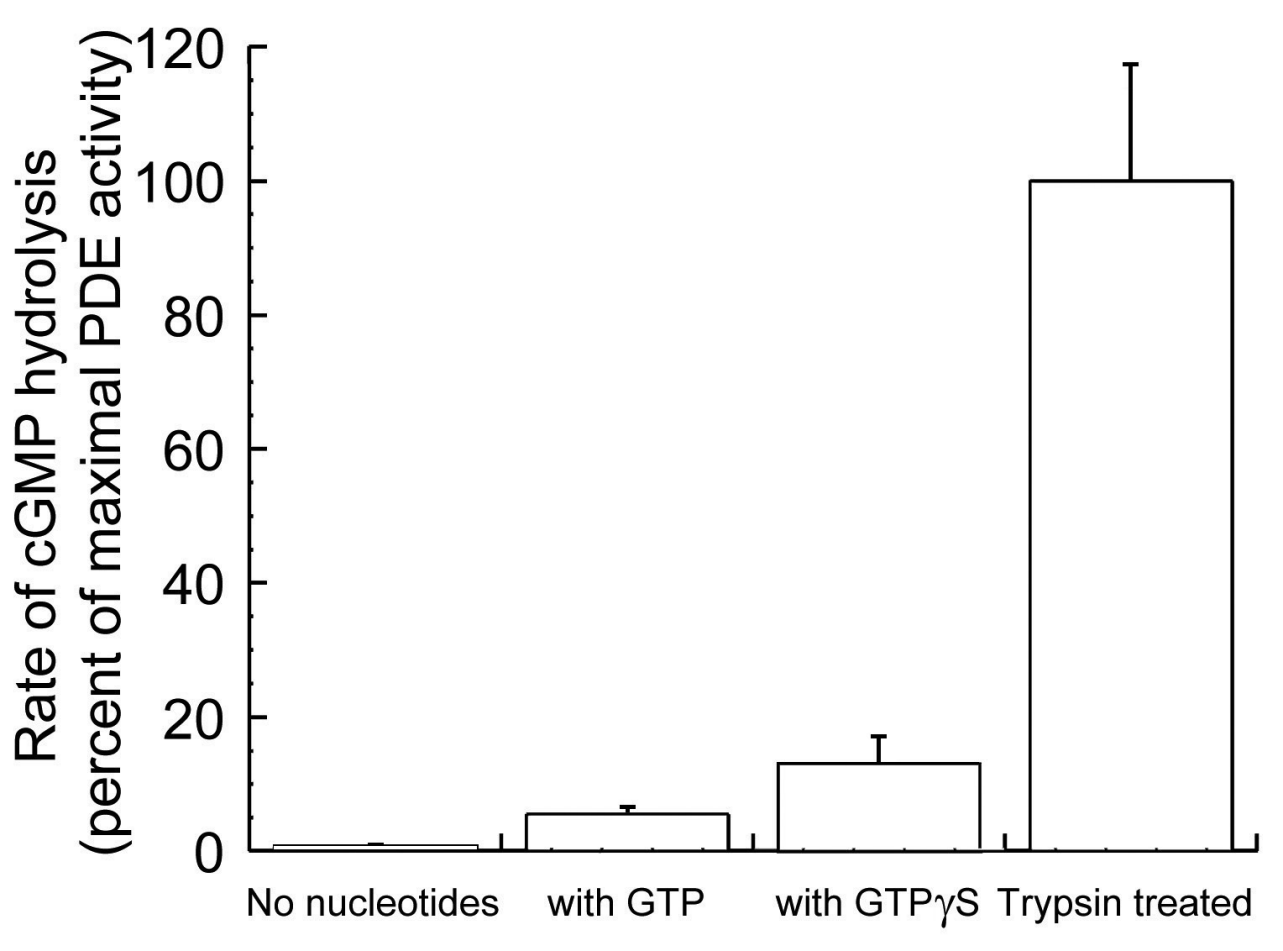
PDE enzymatic activities in isolated rod outer segment. Phosphodiesterase (PDE) activities were measured by thin layer chromatography using [8-^3^H] cGMP as the hydrolysis substrate. Rod outer segment (ROS) from dark-adapted rats were isolated, permeabilized, mixed with either 0.5 mM GTP, 0.5 mM GTPγS, or no nucleotide before they were exposed to light. Maximal PDE activity in ROS was elicited by trypsin activation (p≤0.001 for all groups).

To examine possible links between PDE translocation and PDE enzyme activity in ROS, we measured PDE activity in isolated ROS by using thin layer chromatography as previously described with [8–^3^H] cyclic GMP as the substrate [29]. Trypsin proteolysis was optimized using a range of concentrations and time periods to obtain maximal PDE activation (data not shown). PDE activity with GTPγS was about 15% of the maximal levels achievable with trypsin activation (Figure 5). These data suggest that, in permeabilized ROS, only an estimated 15% of total PDE molecules were activated even in the presence of saturating light and GTPγS, since similar K_cat_ were reported with trypsin- and G_tα_/GTPγS-activated PDE [15,30].

## Discussion

Previously, a uniform distribution of PDE over the rod disc membrane surface was reported using enzyme-based cytochemical methods [22,23,27]. In these reports, exogenous or endogenous nucleotidases were used to dephosphorylate 5′ GMP, the product of PDE enzyme activity, and resulting phosphate ions were visualized as lead precipitates. The appropriateness of this method for precise localization for PDE is questionable, since un-reacted phosphate ions are highly soluble and can readily diffuse in the ROS cytosol until they are precipitated by the lead ions. In a later study, immunogold EM with PDE antibodies revealed a random distribution of PDE over the ROS disc membranes in light-adapted retinas [26], in agreement with our results. However, a critical experiment using dark-adapted photoreceptor disc membranes was not undertaken, and thus the effects of light on the subcellular distribution of PDE were not observed.

In this study, we used immunogold electron microscopy to characterize light-induced translocation of PDE on rod disc membranes. Neither conventional light microscopy nor confocal fluorescence microscopy provides sufficient spatial resolution. In contrast, the immunogold EM labeling method used here provides spatially detailed, semiquantitative data on protein abundance within morphologically identifiable subcellular domains [31,32]. In addition, this approach has permitted localization of PDE in dark-adapted photoreceptor disc membranes, since immunogold EM does not rely on the presence of light-induced PDE activity for effective PDE labeling.

A principal conclusion from the present study is that light alters radial distribution of PDE on rod disc membranes. The change in PDE distribution is subtle yet statistically significant. To ensure the accuracy of the results, only the most central longitudinal sections of each ROS were selected for quantification. In a ROS domain within 25 nm of the disc edge, PDE concentration was significantly higher in dark-adapted retina—about twice that found in light-exposed samples (p≤0.002**)**. In contrast, the distribution of PDE in light-adapted rod is similar to a simulated random distribution (p>0.9). This relatively random distribution of PDE in light-adapted disc membranes agrees with earlier histocytochemical studies relying on the enzymatic activity of PDE [22,23,27]. The light-dependent translocation of PDE was almost complete within 3 min of light exposure, faster than ROS to rod inner segment (RIS) translocation of transducin [8], even considering the delay that may have been caused by the speed of fixative penetration. GTP is necessary for this light-induced movement of PDE, but GTP supplementation alone in the absence of light did not produce the movement. Moreover, replacement of GTP by GTPγS prevented the light-induced movement of PDE, suggesting that both light and hydrolysable GTP are required for the translocation of PDE.

The molecular mechanism that governs the radial movement of PDE is still unclear. One could hypothesize that the movement of PDE could be influenced by direct interaction with the rim specific protein glutamic-acid-rich protein (GARP2), an inhibitor of PDE activity [26]. GARP2 is a product of alternative splicing of the β-subunit of the rod cGMP-gated ion channel and is postulated to bind non-activated PDE and therefore lower the “dark noise” by regulating spontaneous activation of PDE [33]. It is conceivable that PDE is more concentrated near disc rim in the dark-adapted rod through binding with GARP2, since similar direct interaction of PDE with plasma membrane cation channels was previously reported [24,25]. However, the observation that GTPγS prevents PDE movement away from disc rim could not be easily explained by binding to GARP2.

Although our data do not rule out other possibilities, position of PDE on the disc membrane might also reflect alterations of direct lipid-protein interactions. PDE interacts with a large number of phospholipids present in the disc membrane, and this association is dependent upon the activated state of PDE [34]. Transducin is an example of such lipid interactions, which profoundly influence its cellular localization and the kinetics of rod photo-response [35].

**Figure 6:**
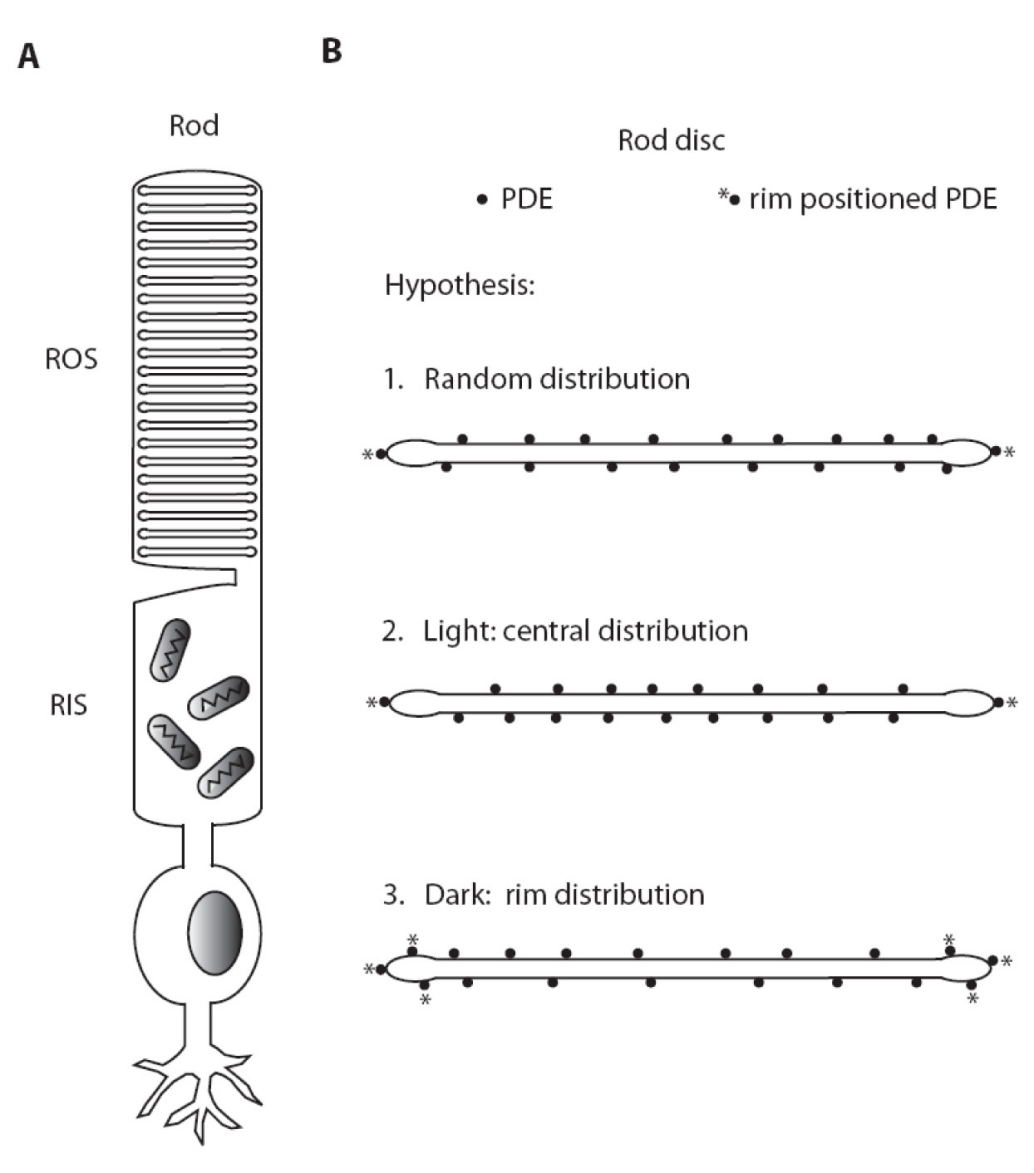
Schematic representation of light-induced PDE translocation on rod outer segment disc membrane. Panel **A** shows schematic drawing of photoreceptor with specialized rod outer segment domain containing stacks of discs embedded with visual signaling proteins of rhodospin, transducin, and phosphodiesterase (PDE). Abbreviations: rod outer segment (ROS); rod inner segment (RIS). Panel **B** illustrates enlarged ROS disc membranes with PDE molecules showing a hypothesized random distribution of PDE molecules. Light induces a centripetal translocation of PDE away from the rim-concentrated distribution in dark-adapted states.

In summary, our results clearly demonstrate a previously unreported light and GTP-dependent radial movement of PDE molecules on ROS disc membranes (Figure 6). Localization of PDE at the rim region might provide a functional advantage in reducing cGMP concentrations around the vicinities of the cGMP dependent ion channels, resulting in rapid ion channel closure in response to light stimulus. Moreover, the observed light-dependent migration of PDE away from the disc edge may contribute to one element of downregulation of rod sensitivity to light by reducing the number of PDE molecules available in those functionally advantageous positions. This position-related downregulation of light sensitivity might be further amplified if it should be confirmed that the association of PDE with the rim-specific sites is a prerequisite for its activation by G_tα_-GTP. Our observation that the fraction of rim-positioned PDE was quite comparable to the fraction of maximal PDE activity suggests the possibility that light- and GTP-dependent PDE activation might occur only within a specialized disc rim region but not in the center of discs. Although evidence provided in this report is in agreement with these hypotheses, they are far from conclusive. Further study is required to determine the exact mechanism and functional significance of light-mediated translocation of PDE in the ROS disc membrane.
